# Recent Advancements in the LC- and GC-Based Analysis of Malondialdehyde (MDA): A Brief Overview

**DOI:** 10.1007/s10337-012-2237-1

**Published:** 2012-04-08

**Authors:** Martin Giera, Henk Lingeman, Wilfried M. A. Niessen

**Affiliations:** 1 Biomolecular Mass Spectrometry Unit, Leiden University Medical Center (LUMC), Albinusdreef 2, 2300 RC Leiden, The Netherlands; 2 BioMolecular Analysis, VU University Amsterdam, De Boelelaan 1083, 1081 HV Amsterdam, The Netherlands

**Keywords:** Malondialdehyde (MDA), Derivatization, LC–MS(MS), Biomarker

## Abstract

Malondialdehyde (MDA) is an end-product of lipid peroxidation and a side product of thromboxane A_2_ synthesis. Moreover, it is not only a frequently measured biomarker of oxidative stress, but its high reactivity and toxicity underline the fact that this molecule is more than “just” a biomarker. Additionally, MDA was proven to be a mutagenic substance. Having said this, it is evident that there is a major interest in the highly selective and sensitive analysis of this molecule in various matrices. In this review, we will provide a brief overview of the most recent developments and techniques for the liquid chromatography (LC) and gas chromatography (GC)-based analysis of MDA in different matrices. While the 2-thiobarbituric acid assay still is the most prominent methodology for determining MDA, several advanced techniques have evolved, including GC–MS(MS), LC–MS(MS) as well as several derivatization-based strategies.

## Introduction

Malondialdehyde (MDA) is an end-product of the radical-initiated oxidative decomposition of poly-unsaturated fatty acids and, therefore, it is a frequently measured biomarker of oxidative stress [1]. More information about reactive carbonyls and proposed intermediates can be found in [2]. In addition, MDA is generated as a side product of thromboxane A_2_ synthesis [3] and by gamma irradiation of DNA [4]. In case of thromboxane A_2_ synthesis, MDA is formed during the conversion of the endoperoxide prostaglandin H2 (PGH_2_) by thromboxane synthase [5]. PGH_2_ is derived from arachidonic acid by conversion via cyclooxygenases [6].

**Fig. 1:**
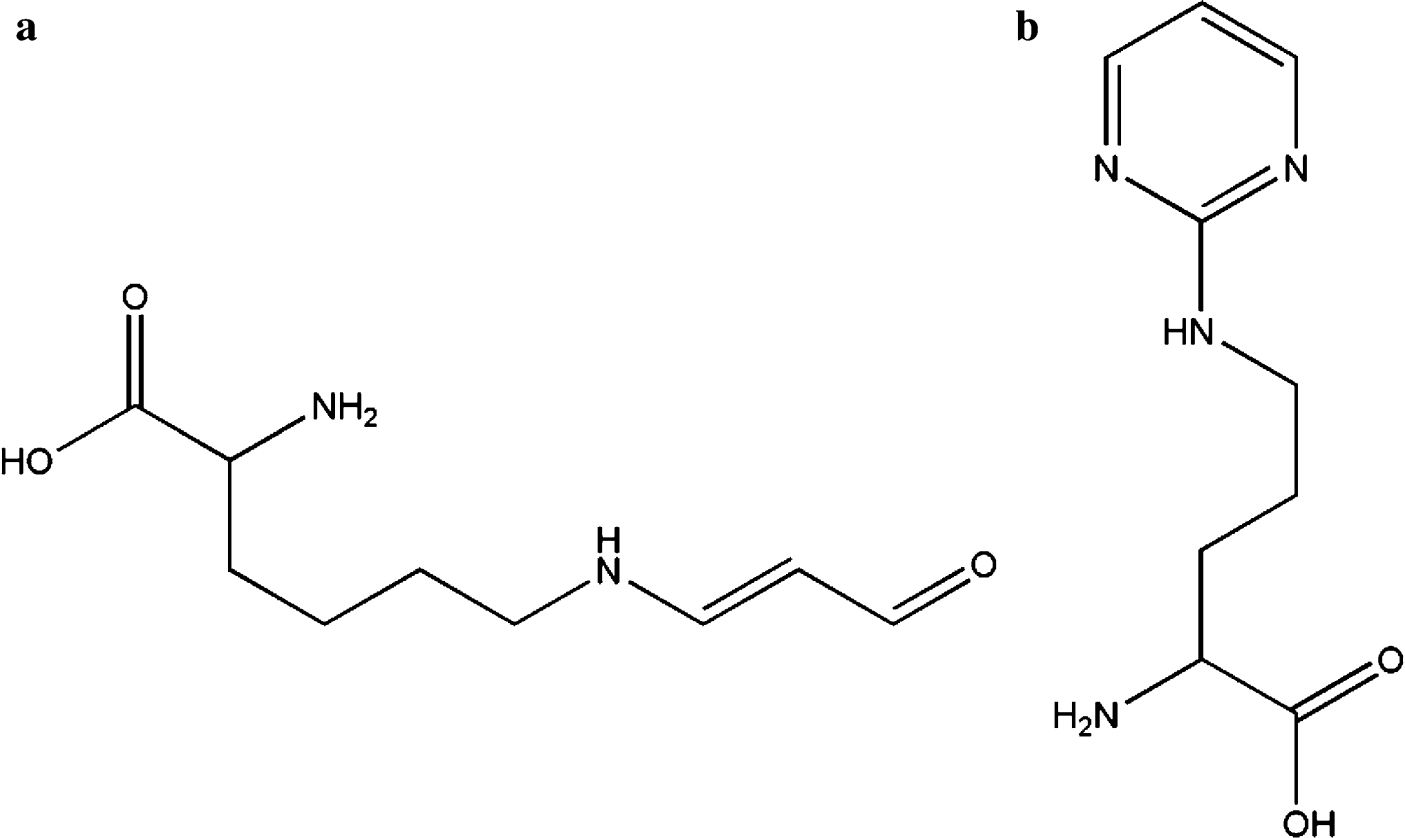
MDA adducts of **a** lysine–*N* ^ε^-*β*-lysyl-aminoacrolein and **b** arginine–*N* ^δ^-(2-pyrimidyl)-l-ornithine [10, 11] as MDA adduct formation mainly occurs on the protein level as shown in the α-amino carboxylic acid group would be incorporated into a peptide bond

Particularly, MDA’s high reactivity and capability of forming adducts with multiple biological molecules such as proteins or DNA has attracted major attention over the last decades [7, 8]. MDA’s high reactivity is mainly based on its electrophilicity making it strongly reactive toward nucleophiles, such as basic amino acid residues (i.e., lysine and histidine). This reactivity is not only based on MDA’s aldehydic nature but is also influenced by its 1,3-dialdehydic structure making it possible to form mesomerically stabilized Schiff bases. As will be discussed below, especially this high reactivity towards nucleophilic molecules has been extensively exploited during the last years to develop several derivatization strategies for MDA. Furthermore, MDA’s high reactivity has for example also been used for the controlled modification of basic amino acids in proteomics studies [9]. As examples the structure of a meta-stable lysine and a stable arginine adduct with MDA is shown in Fig. 1.

However, not only in the life sciences field but also in food analysis, MDA serves as a biomarker of lipid oxidation (oxidative decomposition) [12, 13]. MDA’s potential mutagenicity, atherogenicity and cancerogenicity [14] additionally underline the importance of monitoring this substance.

Taken together, these facts point out that MDA is not simply a biomarker for the oxidative decomposition of poly-unsaturated fatty acids but instead might also be derived from other sources and more importantly shows physiological relevance as it is capable of forming DNA and protein adducts [8, 15].

Several different strategies have been pursued for measuring MDA in a variety of different matrices (i.e., plasma, urine, and saliva). The employed analytical strategies can roughly be sub-divided into derivatization-based and label-free methodologies. Particularly for the analysis of biological matrices, these strategies have been coupled to separation techniques such as liquid chromatography (LC) or gas chromatography (GC). Due to its high reactivity, the majority of present MDA might be bound to plasma proteins and other species. Hence, it is important to emphasize whether the free or total MDA level is determined. Referring to this fact, it is also essential to choose suitable sample preparation conditions particularly hydrolysis conditions when total MDA is to be analyzed and to consider the release and/or formation of MDA when harsh derivatization conditions are to be applied [16]. An overview about selected analysis techniques for MDA is given in Table 1.

**Table 1:** Overview of LC- and GC-based methods for the measurement of MDA

Detection technique	Separation technology	Derivatization reagent (derivatization time)	Matrix	Sample preparation	LOD	Comment	References
UV	RP–LC (C_18_)	–	Serum (S)	Protein precipitation with HClO_4_	12 nM	Total MDA levels (bound and unbound MDA)	[20]
UV	RP–LC (C_18_)	Diaminonaphthalene (180 min)	Plasma (P) S	Protein precipitation (metaphosphoric acid)	0.05 nM	Free and total MDA levels	[32]
UV	RP–LC (C_18_)	DNPH (60 min)	Urine (U)	Liquid liquid extraction (LLE) after derivatization	56 nM	Free MDA levels, standard addition quantitation	[23]
UV	RP–LC (C_18_)	DNPH (10 min)	P	Protein precipitation with HClO_4_, LLE after derivatization	2.1 μM	Total MDA levels (bound and unbound MDA)	[27]
MS(MS)	RP–LC (porous graphite—Hypercarb)	–	Exhaled breath condensate (EBC) U P	EBC and U—SPE, P–protein precipitation	0.4 nM (EBC) 1.3 nM (U) 0.4 nM (P)	Free MDA levels	[22]
MS	RP–LC (C_18_)	4-APC (240 min)	P U	P–Protein precipitation (ACN) On-line SPE (WCX)	0.5 nM (standard solution)	Free MDA levels	[36]
MS(MS)	RP–LC (C_18_)	DNPH-d_3_-DNPH (120 min)	EBC	–	n.d.	AIDA quantification	[34]
FD	RP–LC (CN)	2-Aminoacridone (90 min)	U	–	1.8 nM	Free MDA levels, standard addition quantification	[33]
FD	RP–LC (C_18_)	TBA (60 min)	U P	LLE after derivatization	128 nM	Total MDA levels (free and bound MDA)	[25]
FD	RP–LC (C_18_)	Dansylhydrazine	Tissue homogenate (mouse)	Automated SPAD	0.02 μg/mL	Automated SPAD system (standard addition quantification)	[30]
FD	RP–LC (C_18_)	FMOC-hydrazine (240 min)	P	Protein precipitation (ACN)	4.0 nM	Free and total MDA levels	[28]
MS	GC	Pentafluorophenylhydrazine (10 min)	U	Head space injection	0.56 nM	–	[37]
MS (SIM)	GC	Phenylhydrazine (30 min)	P Microsomes	LLE with or without previous hydrolysis/protein precipitation	5 pmol injected	Free and total MDA levels	[16]
MS	GC	2,2,2-Trifluoroethylhydrazine (40 min)	Blood	Head space–SPME	5.6 nM	–	[38]
NPD	GC	Methylhydrazine (60 min)	Cod liver oil treated with Fenton’s reagent	Head space–SPME	0.01 μM	–	[39]
ECD	GC	TCPH (60 min)	P (defatted)	LLE after derivatization + drying step over sodium sulfate	0.05 μM	–	[40]
ECD	GC	*O*-(2,3,4,5,6-pentafluorbenzyl)hydroxylamine (60 min)	U (rat)	LLE after derivatization + drying step over sodium sulfate	50 fmol	Total MDA	[43]

*n.d.* Not determined, *SIM* selected ion monitoring, *RP* reversed phase

## Analysis of Malondialdehyde

### Label-Free Analysis

Several possibilities for the label-free analysis of MDA exist, ranging from simple UV-based methodologies to LC–MS(MS) platforms.

**Fig. 2:**
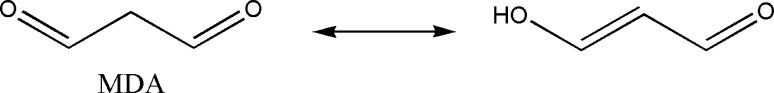
Mesomeric structures of MDA

Malondialdehyde has a p*K*_a_ value of approximately 4.5 and a melting point of around 72 °C [17]. The rather low p*K*_a_ value of MDA can be explained from its mesomeric structure (Fig. 2). Basically, MDA can be drawn either as a di-aldehyde or as a vinylogenic carboxylic acid to explain the low p*K*_a_ value observed for this substance. Based on its mesomeric structure, which possesses a *α,β*-unsaturated carbonyl function, MDA can spectrophotometrically be determined under acidic conditions at 245 nm or under alkaline conditions at 267 nm [18, 19].

It is evident that such an ultraviolet (UV) absorbance-based methodology carried out at low wavelengths suffers from rather poor sensitivity and selectivity, especially when biological matrices are involved. Hence, separation techniques such as reversed-phase liquid chromatography (LC) [20] or capillary electrophoresis (CE) [21] have been used to separate MDA from matrix constituents and to facilitate its detection by UV-absorbance. While Karatas et al. [20] used perchloric acid (HClO_4_) for protein precipitation and the release of bound MDA in human serum samples, Wilson et al. [21] applied a combined centrifugation, filtration approach to prepare plasma samples for analysis.

**Fig. 3:**
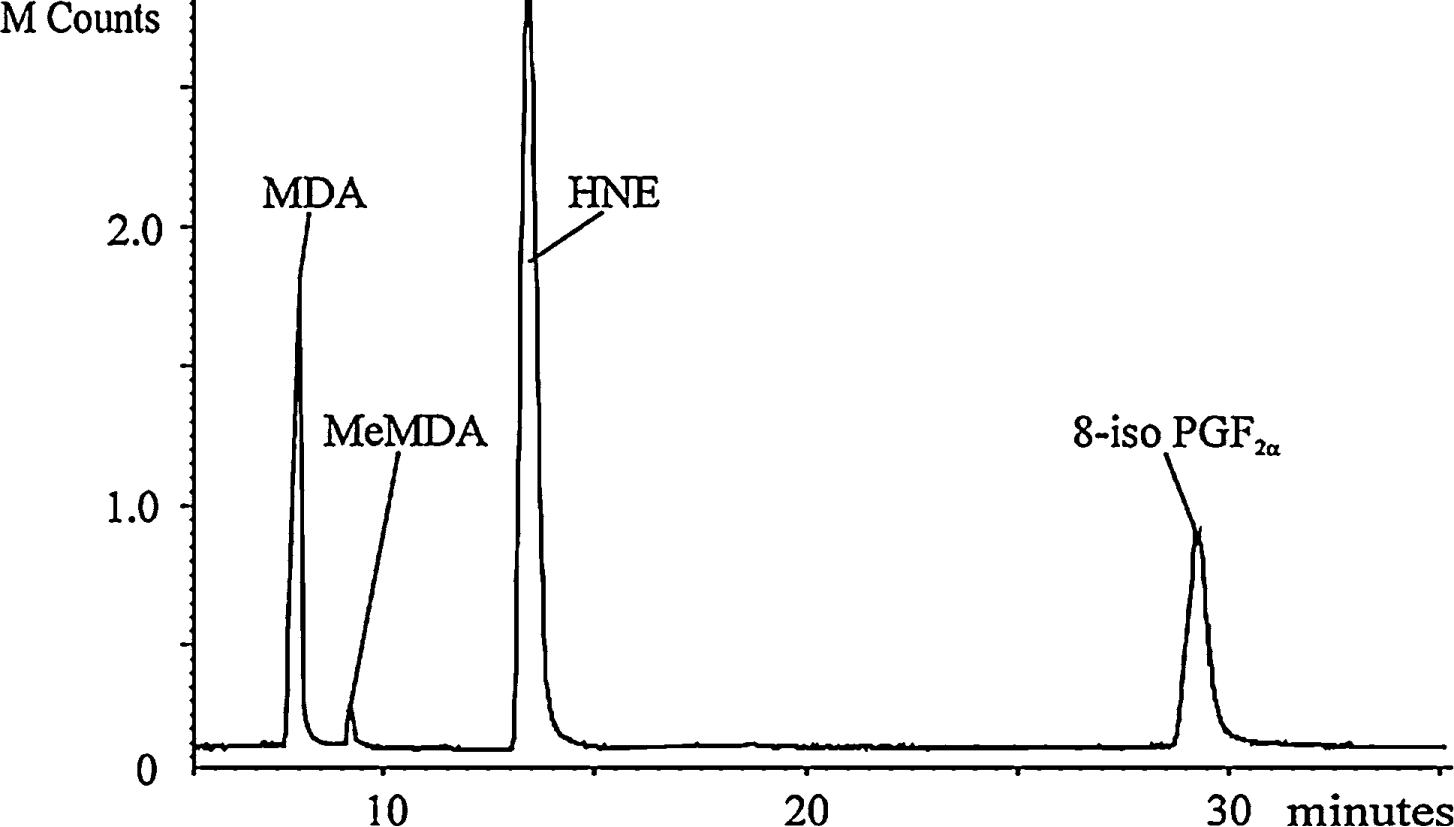
Chromatogram of a urine sample obtained with the label-free LC–MS/MS method, described by Syslová et al. [22] (taken with permission from [22]). *MDA* malondialdehyde, *MeMDA* methylmalondialdehyde, *HNE* hydroxynonenal, *8-iso PGF*_*2α*_ 8-iso prostaglandin F2α

An LC–MS(MS)-based methodology employing a triple-quadrupole mass spectrometer (MS) and a porous graphite column (Hypercarb) for the measurement of MDA and other biomarkers of oxidative stress in exhaled breath condensate, urine and plasma was recently published by Syslová et al. [22]. The method showed very low limits of detection (LOD) for all mentioned matrices and can be applied without the necessity of labeling MDA. Although the method of Syslová et al. did not demand any derivatization allowing the detection of MDA, solid-phase extraction (SPE), incorporating methylmalondialdehyde as internal standard, was applied to exhaled breath condensate (EHB) and urine samples, while plasma samples underwent protein precipitation as sample pretreatment. Limits of detection (LOD) as low as 32 pg/mL (0.4 nM) in exhaled breath condensate could be reached. A representative chromatogram of a urine sample is shown in Fig. 3.

### Derivatization-Based Methodologies

The vast majority of analytical approaches for the determination of MDA make use of some kind of derivatization. Most strategies are based on the “aldehydic-reactivity” of MDA, hence employing hydrazine-based derivatization reagents.

**Fig. 4:**
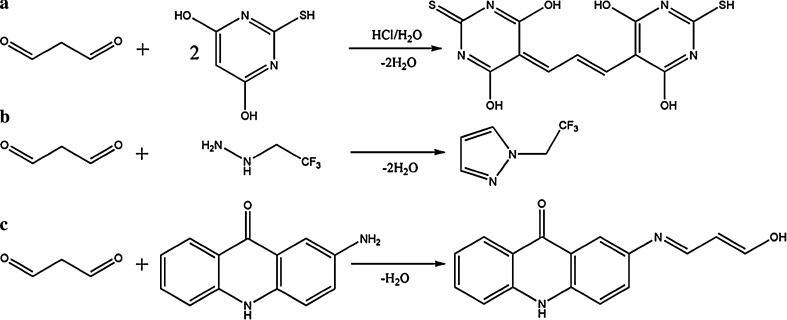
Reaction of MDA with different derivatization reagents, **a** TBA assay, **b** labeling with 2,2,2-trifluoroethylhydrazine, **c** labeling with 2-AA (reprinted with permission from [33, 45]

Still, the most frequently applied technology is the “classic” 2-thiobarbituric acid (TBA) assay [23] in which two molecules of TBA condense with one molecule of MDA to give a colored reaction product (cf Fig. 4), which can be measured spectrophotometrically at 535 nm, or by fluorescence detection with excitation at 530 nm and emission at 550 nm. The TBA assay intrinsically is not specific for MDA [24], and therefore, it is frequently combined with LC separation and fluorescence detection of the formed products [25]. Still, the TBA assay including the LC step was reported to overestimate urinary MDA concentrations by almost tenfold. This might be related to the harsh conditions (100 °C, acidic conditions) needed to yield the colored reaction product. In a recent study, examining the inter-laboratory variation in the measurement of oxidative stress biomarkers, which was mainly accomplished by the combined LC-TBA assay, Breusing et al. [26] could show huge variations between the actual measured MDA levels in irradiated human plasma samples. Surprisingly, in this study the LC-TBA assay proved to give the lowest MDA levels when compared to other assay formats.

#### LC–UV/FD Analysis

Due to the drawbacks of the classic TBA assay being non-specific for MDA and not allowing a distinction between free and bound MDA, several novel approaches for the specific determination of MDA have been developed. Especially hydrazine-based derivatization reagents, such as dinitrophenylhydrazine (DNPH) [27], FMOC-hydrazine [28], or dansylhydrazine [29], have been employed in the LC analysis of MDA. While DNPH has been used in combination with UV detection, the later reagents were used for the fluorescence detection of the resulting derivatives. All of these reagents have been used to determine MDA in human plasma, DNPH has additionally been applied to determine MDA in human urine [23]. Dansylhydrazine in combination with a so-called solid-phase analytical derivatization (SPAD) where the derivatization is carried out on a solid support has also been applied for the analysis of MDA in mouse tissue homogenates [30].

Sample preparation in the cited DNPH-based method [27], included alkaline hydrolysis for MDA release, followed by a HClO_4_-based protein precipitation and derivatization. Finally, the derivatives had to be extracted with hexane, dried and reconstituted before analysis. Another study involving the DNPH reagent to investigate urinary MDA levels [23], described a combined derivatization extraction step, involving pentane as the extraction solvent. Especially for the preparation of the derivatization reagent solution, the authors describe a rather elaborate protocol, including chloroform extractions subsequent to preparing the reagent solution in 4 N HCl. In case of the FMOC reagent [28], free MDA could be analyzed by protein precipitation with subsequent derivatization prior to analysis. A more automated extraction and derivatization protocol is presented in the SPAD-based analysis platforms [29, 30], which have been used to determine MDA in tissue homogenates as well as plasma samples. Although the SPAD analysis is a fully automated process, its complexity might be a complication for its applicability to large sample cohorts.

Three non-hydrazine-based reagents which were used for the determination of MDA are: 1-methyl-2-phenylindole [31], which is a colorimetric reagent, forming a chromophore with a UV absorbance maximum at 586 nm. Diaminonaphthalene [32], which was used for the LC-UV based determination of free and bound MDA in human plasma samples, and 2-aminoacridone (2-AA) [33], which was used for the LC–fluorescence detection (FD) of free MDA in human urine.

In the case of methyl-2-phenylindole, the reagent is directly added to the sample of interest (i.e., plasma and tissues homogenate), the mixture is incubated and centrifuged before the absorbance is read. In the case of diaminonaphthalene, protein precipitation with or without release of MDA from proteins was applied. The authors described the limit of detection for their method to be better than 50 pM being very low for a UV-based methodology [32]. Urine samples derivatized with 2-AA did not undergo any sample pretreatment. In case of the 2-AA reaction, the formed derivative showed distinct changes in the conjugated π-system, and, respectively, in its UV-absorbance as well as fluorescence properties, thereby possibly allowing the development of a specific plate reader-based assay (see Fig. 4). Other advantages of this methodology are: (a) the simple derivatization protocol, which basically consists of mixing the urine sample with citrate buffer and reagent solution followed by an incubation step (90 min) and (b) the possibility of isocratic analysis, thereby allowing a rather high throughput as sample analysis times in this case were described to be less than 4 min [33].

#### LC–MS(MS) Analysis

Besides the fact that most MDA derivatives are analyzed by LC–UV or LC–FD analysis, also LC–MS(MS)-based strategies involving a derivatization procedure have been described. One such approach uses DNPH and deuterium-labeled d_3_-DNPH to apply a novel isotope dilution technique called alternate isotope-coded derivatization assay (AIDA) for the determination of MDA and other biomarkers of oxidative stress in exhaled breath condensate [34]. Another reagent used for the LC–MS detection of MDA and other aldehydes is 4-(2-(trimethylammonio)ethoxy)benzenaminium halide (4-APC), which is coupled via reductive amination to the aldehyde group [35]. This reagent was used in combination with an on-line weak cation exchange (WCX) solid-phase extraction LC–MS system, for the detection of MDA and other aldehydes in urine and plasma [36]. In case of urine samples, a simple centrifugation step was carried out as sample pretreatment, while plasma samples underwent protein precipitation with acetonitrile before being subjected to derivatization and subsequent analysis by on-line SPE LC–MS [36].

#### Gas Chromatographic MDA Analysis

As for the LC-based determinations, hydrazine labels dominate the employed strategies for the GC-based methodologies as well. Phenylhydrazine has been used to analyze MDA in plasma and microsomal samples [16], as due to very mild reaction conditions free as well as total MDA levels can be determined. Pentafluorophenylhydrazine has been used for the head space-based analysis of MDA in urine [37]. A combined head space–solid phase micro extraction (SPME) approach for the determination of MDA in human blood incorporated trifluoroethylhydrazine as derivatization reagent [38] (see Fig. 4). Along these lines also methylhydrazine has to be named, which was used for the GC–nitrogen phosphorus detector (NPD)-based detection of MDA in various matrices [2, 39]. Another hydrazine-based label which was used in combination with an electron capture detector (ECD) is 2,4,6-trichlorophenylhydrazine (TCPH) [40]. The reagent was developed for the determination of total MDA in urine samples [41].

Two non-hydrazine-based reagents which have been used for the analysis of MDA by GC–MS(MS) are pentafluorophenylbromide [42] and *O*-(2,3,4,5,6-pentafluorbenzyl)hydroxylamine hydrochloride [43]. Pentafluorophenylbromide yields a doubly derivatized species, which was analyzed via GC–MS(MS) analysis in the selected reaction monitoring mode (*m*/*z* 253 → 177 and *m*/*z* 251 → 175). The second reagent, *O*-(2,3,4,5,6-pentafluorbenzyl)hydroxylamine was used to determine MDA in rat urine by a GC–ECD based method [43].

In the case of plasma samples, Cighetti et al. [16] applied a liquid–liquid extraction (LLE) employing hexane after derivatization, with or without previous protein precipitation using acetonitrile (ACN). In case of microsomal incubations, an additional filtration step was applied. Shin et al. as well as Fujioka and Shibamoto could successfully circumvent the need of protein precipitation or LLE in urine, blood and cod liver oil samples by employing head space or head space SPME analysis after MDA derivatization [37–39]. When the TCPH reagent was applied to defatted plasma samples, the sample preparation involved LLE and a drying step over anhydrous sodium sulfate before subsequent drying under nitrogen was carried out [40]. Dreissigacker et al. [42] employed a toluene extraction of the dried residue after derivatizing whole plasma samples. De Zwart et al. [43] analyzed total MDA after acid hydrolysis in rat urine samples. After derivatization, LLE employing heptane and a drying step over sodium sulfate were applied.

## Conclusions

Malondialdehyde has been analyzed in a wide variety of different matrices in numerous studies dealing with oxidative stress parameters or the modification of endogenous substances such as proteins, or DNA. Several technologies for the determination of free and total MDA which are more sensitive and selective compared to the classic TBA assay have been developed during the last decade. Although the superior performance of all these methods has been proven, the TBA assay is still widely applied, even though its unspecific nature is known today. The most likely reason for this might be its convenient utilization in particular to large sample numbers, needing only a plate reader instrument. Hence, it seems to be crucial to develop a selective and sensitive derivatization strategy for MDA, which can easily be applied to large sample cohorts. Such a reagent could possibly evolve from a non-hydrazine-based reagent, such as 2-aminoacridone, where the coupling of MDA causes distinct changes in the spectroscopic properties and thereby might lead to a selective plate reader-based detection of this important substance. Another strategy to develop such a plate reader-based analysis platform might be the development of Förster resonance energy transfer (FRET)-based derivatization strategies [44] for MDA. Such reagents could possibly be developed on the basis of the aromatic amine binding properties of MDA (cf 2-AA), changing the spectroscopic properties of a primary fluorescent molecule, now allowing FRET, involving a secondary fluorescent part of the reagent. Additional possibilities for the development of advanced detection technologies for MDA might also arise from so-called f-trap reagents, which due to their fluorine containing nature allow the selective removal of excess reagent via fluorous solid phases [44].

The method of Syslová et al. [22] offers a highly sensitive analysis method for MDA, which does not require a derivatization step. The main drawback of the method is the need for highly expensive equipment, such as the employed triple-quadrupole MS. Within the derivatization-based methods, the GC-(head space) SPME methodologies and the 2-AA based method for urine samples seem to be superior as they demand minimal sample pretreatment and show low limits of detection.
